# Prioritization of Ethical Themes When Surrogates Object to Technology Removal After Brain Death Determination

**DOI:** 10.1007/s12028-025-02390-2

**Published:** 2025-10-08

**Authors:** Elena Montag, Matthew P. Kirschen, Pamela Nathanson, Wynne Morrison, Evan Fieldston, Jennifer K. Walter

**Affiliations:** 1 Department of Pediatrics, Children’s Hospital of Philadelphia, Philadelphia, PA USA; 2 Department of Anesthesiology and Critical Care Medicine, Children’s Hospital of Philadelphia, Philadelphia, PA USA; 3 Department of Medical Ethics, Children’s Hospital of Philadelphia, Philadelphia, PA USA; 4 Department of Medical Ethics and Health Policy, University of Pennsylvania School of Medicine, Philadelphia, PA USA

**Keywords:** Brain death, Death by neurological criteria, Ethics, Family, Removal of technologic support, End-of-life, Uniform Determination of Death Act, Hospital policy

## Abstract

As hospitals revise their policies for brain death/death by neurologic criteria (BD/DNC), they should provide transparent and clear plans for how to handle surrogate requests to continue technology after declaration of death. National guidelines provide little guidance in this matter, and individual institutions should make decisions grounded in an understanding of their local constraints and the ethical values at stake. Ethical arguments exist in favor of and opposed to allowing continuation of technologic support for families who object to BD/DNC. Prioritization of these ethical values influences how institutions handle objections to removal of technologic support after BD/DNC determination. A narrative review of the literature identified six ethical values relevant to institutional responses to surrogate objections: inappropriate treatment, distributive justice, BD/DNC as philosophical or legal death not biological death, dignity and respect, surrogate authority, and medical mistrust. We articulate three potential paths forward an institution can take and the relative prioritization of ethical values in each case. When developing or revising hospital policies on BD/DNC, hospitals must understand the ethical values in tension with any particular approach taken. Hospitals vary in the populations they care for and the resources at their disposal, which may impact prioritization of ethical principles at stake in these decisions. Although some ethical values may not be prioritized, institutions should design systems to honor them to the extent possible given the plurality of beliefs and experiences of the patients for whom we care.

**Supplementary Information:**

The online version contains supplementary material available at 10.1007/s12028-025-02390-2.

## Introduction

The criteria for determination of brain death/death by neurologic criteria (BD/DNC) were established in 1968 by the Ad Hoc Committee at Harvard Medical School and formed the basis for model state law in 1981, the Uniform Determination of Death Act [1]. Diagnostic criteria for BD/DNC have been revised several times, most recently in 2023 through a consensus process involving several professional medical societies [2]. BD/DNC is defined in clinical practice in the most recent guidelines as permanent loss of all function of the brain after catastrophic neurologic injury, resulting in permanent loss of consciousness, brainstem areflexia, and lack of spontaneous respiratory effort to a hypercarbic and acidotic challenge [3]. There has been debate about the biological and scientific validity of the “whole brain” concept of BD/DNC, which resulted in an attempt to revise the language of the Uniform Determination of Death Act. This effort was abandoned due to lack of consensus and concern regarding whether it can be enacted.

Despite more than 50 years of experience with BD/DNC, many clinicians report encountering objections by families to evaluation of their loved one for BD/DNC or removal of technologic support after BD/DNC declaration [4]. In an analysis using VPS, a pediatric intensive care unit (ICU) database representing more than 200 units, 2.7% of patients remained in the ICU at least 48 h after they were declared BD/DNC and 0.7% of patients remained after 5 days. We use the term “technologic support,” acknowledging that others use “organ support” and “somatic support” instead. Sixty-one percent of US pediatric neurologists and intensivists and 47% of adult neurologists have been asked by surrogates to continue technologic support after BD/DNC declaration [4, 5].

Legal rulings have increased in heterogeneity over recent years as challenges to BD/DNC have become more frequent. Legal arguments include that the medical criteria used to diagnose BD/DNC fail to meet legal requirements for death declaration, that consent should be required prior to BD/DNC evaluation, and that accommodation for religious and moral objections to BD/DNC should be universal [6]. Litigation may be leading to further variability in clinical practice, institutional policies, and state laws regarding handling of BD/DNC objections [6].

How health systems operationalize care for patients whose surrogates refuse removal of technologic support varies, and guidelines provide limited guidance. One survey found 81% of adult neurologists in states that mandate accommodation for religious beliefs after BD/DNC declaration and 66% in nonaccommodation states reported institutional policies governing removal or continuation of technologic support after BD/DNC declaration [4]. A study of 331 adult hospitals nationwide found nearly 80% of existing protocols included no guidance addressing surrogate objections to technology withdrawal [7]. Among pediatric institutions, only 42% of hospital policies provided such recommendations [8].

This narrative review synthesizes the ethical arguments and principles related to addressing surrogate requests for continued technologic support after BD/DNC declaration. This ethical analysis aims to inform efforts to establish, revise, and standardize hospital policies when navigating these complex clinical scenarios. Many of the ethical arguments related to withdrawal of technology are similar to the arguments that may be made to accommodate or override surrogate objections to even performing the testing (including apnea testing), but for this article, we will narrow our focus to how to handle situations for when declaration of death has been made. Institutional ethics committees may play a variety of roles in these situations, depending on institutional culture and resources. In some circumstances, ethics oversight may help produce policies governing these situations; in others, ethicists may sit on oversight committees that review instances of BD/DNC objections; and in some institutions, there may be a mandatory ethics consultation that engages surrogates directly.

## Methods

This narrative review was conducted to identify published articles detailing an ethical analysis of withdrawal of technologic support after diagnosis of BD/DNC.

### Search Criteria

**Fig. 1:**
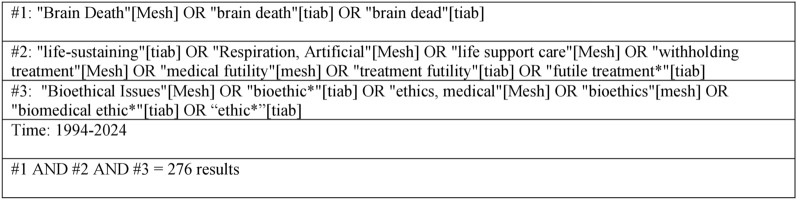
PubMed search criteria

We searched PubMed in April 2024 using Medical Subject Headings and search terms related to BD/DNC, ethics, and organ support (Fig. 1).

### Eligibility

We included publications that discussed “organ support” and/or withdrawal of technology after diagnosis of BD/DNC, along with relevant ethical principles and arguments. Publications were excluded if they focused on pregnancy or support as a bridge to organ donation.

### Data Extraction and Synthesis

Thematic content analysis was conducted using predetermined ethical concepts and areas of importance, while allowing for the addition of relevant thematic elements emerging during the review. This study did not include human subjects research or retrospective data review and therefore did not require institutional review board oversight.

### Process for Prioritization of Ethical Values in Different Approaches

Articulation of the three approaches and the prioritization of the ethical values in each approach was initially proposed by the senior author (JKW). The approaches and the prioritization were then discussed in conjunction with all authors at a special session of the ethics committee when preparing to revise the hospital’s policy on BD/DNC with special attention to making transparent an approach to objections to testing or withdrawal of technology. This discussion allowed for the ordering to be “pressure tested” and vetted by a range of stakeholders across the institution, including many leaders from the ICUs from multiple professions. Iterative discussion and revision of the article allowed for elucidation of the ways in which deprioritized values may still be honored.

## Results

### Article Selection

The PubMed search yielded 276 results. One author (EM) screened the title and abstract of each publication and excluded those published in a language other than English and those primarily focused on cardiorespiratory death, BD/DNC and pregnancy, and organ donation. The full text of all remaining publications (*n* = 63) was then reviewed for relevance. Additional articles (*n* = 12) were identified through review of references. A total of 75 articles were analyzed (Supplementary Table).

### Ethical Themes

We identified six ethical themes concerning the discourse around technologic support for patients declared BD/DNC (Table 1).

**Table 1:** Ethics themes in the discourse around objections to removal of technologic support after BD/DNC declaration

Ethical theme	Explanation	Arguments in favor of continued technologic support in BD/DNC patients	Arguments against continued technologic support in BD/DNC patients
Inappropriate treatment	Administration of medical treatments unable to fulfill a desired physiologic effect	If a patient’s surrogate believes them to be alive, then to deem all further interventions as “inappropriate” fails to account for reasonable accommodation for their medical goals of life extension [10, 20, 26]	Any care provided to a patient declared BD/DNC is by definition inappropriate, as there is no possibility of neurologic recovery, and the patients are legally dead [11, 23, 27]. Staff caring for patients declared BD/DNC report moral distress if care persists beyond a brief period [28]
Distributive justice	Equitable allocation of relatively scarce resources, including funding, medical supplies, and personnel	In practice, ICU beds are rarely unavailable, and objections to BD/DNC are so infrequent that providing a ventilator and other ICU care does not strain the larger medical system [12]. In cases of strain, such patients may be transferred to long-term care facilities to increase availability of ICU beds [13, 29]	The use of medical supplies, providers, and ICU beds for patients declared BD/DNC is unjust when those resources could be used for others who would reap greater benefit [30–32]
BD/DNC as a philosophical and legal definition of death, not biological	Neurologic death as a debated physiologic diagnosis; BD/DNC as a moral, philosophical, and legal definition of death rather than biological	The biological legitimacy of the concept of BD/DNC is questioned because the criteria used to declare BD/DNC do not require proof that all neurons in the brain have died. Patients who meet criteria for BD/DNC can have preserved neurohormonal function that allows for growth, wound healing, menstruation, and even gestation of a fetus [20]. This ambiguity legitimizes moral and religious objections given the current legal definition of death is a social construct [12, 33]	Although some acknowledge arguments questioning the biological validity of existing BD/DNC definitions and criteria, they argue that from a societal perspective, a consistent definition of death is important to avoid scenarios in which patients in the same neurologic state would be considered alive in some jurisdictions and dead in others [11]. Others claim that the current standards for BD/DNC are sufficiently acceptable because they indicate if not biological death, then permanent loss of consciousness and essential personhood, which is sufficient to declare moral and legal death [16, 25]
Dignity and respect	Honoring the value intrinsic to each human person; accommodating and balancing the stated perspectives of both the patient and the patient’s family to best honor their needs, beliefs, and worth	Respect for the dignity of both the patient and the family requires adhering to their wishes and beliefs about death and providing care their surrogates state they would want [13, 33, 34]	Any further interventions after BD/DNC declaration constitute desecration of a deceased body’s dignity and a violation of the goals of the medical profession. Staff caring for patients and families may experience moral distress if they are asked to provide care that is inconsistent with their perception of treating a patient with dignity and respect [11, 28, 30]
Surrogate authority	The right to independent decision-making of surrogates to reflect a patient’s beliefs and wishes; weighed against the judgment and authority of health care providers in care decisions	The rights of surrogates to make decisions regarding withdrawal of technologic support after BD/DNC declaration and their beliefs about what constitutes death should not be violated [12, 13, 17]	The rights of surrogates are not absolute, especially when their requests are contrary to the best interest of the patient. Surrogate autonomy cannot force a medical provider to continue interventions that are inappropriate or contrary to the guiding principles of medicine [18, 19, 35, 37]
Medical mistrust	Lack of trust in the health care system and providers to care for patients’ interests; the belief that the medical system will provide differential care or act with ill intent toward certain patients	Denying requests for ongoing technologic support increases distrust, particularly when experienced by vulnerable and historically underserved populations, as they may perceive that withdrawal of technology is being pursued to obtain organs or make resources available for other patients [20–22]	Allowing indefinite accommodations exacerbates inconsistency and confusion around death, paving the way for greater controversy and erosion of trust in the medical profession’s ability to accurately determine death [19, 36]

*BD/DNC* brain death/death by neurologic criteria, *ICU* intensive care unit

### Inappropriate Treatment

Inappropriate treatment is defined as interventions that are unable to achieve the desired physiologic goal [9]. For example, performing cardiopulmonary resuscitation on a body displaying signs of rigor mortis would constitute inappropriate treatment. Implicit in the assessment of inappropriate treatment is that physicians have final authority over determining when care is no longer appropriate. Proponents of respecting surrogates’ requests for continued technologic support after BD/DNC declaration argue that if a patient’s surrogate believes them to be alive, then to deem all further interventions as “inappropriate” fails to account for reasonable accommodation for their medical goals of life extension [10]. In contrast, opponents claim that any care provided to a patient declared BD/DNC is by definition inappropriate, as there is no possibility of neurologic recovery and the patients are legally dead [11]. Staff caring for patients declared BD/DNC also report moral distress if care persists beyond a brief period.

### Distributive Justice

The just allocation of limited resources is another point of debate. Those in favor of honoring surrogate objections argue that in practice, ICU beds are rarely unavailable, and objections to BD/DNC are so infrequent that providing a ventilator and other ICU care does not strain the larger medical system [12]. In cases of strain, such patients may be transferred to long-term care facilities to increase availability of ICU beds [13]. Those against continued technologic support argue that the use of medical supplies, providers, and ICU beds for patients declared BD/DNC is unjust when those resources could be used for others who would reap greater benefit [14]. Citing closure of pediatric units across the United States and increasing nursing shortages in adult and pediatric settings, which further diminish the availability of both ICU and long-term care facility beds, those opposed to honoring surrogate requests emphasize the importance of just distribution of the scarce resources [15, 16].

### BD/DNC as Philosophical and Legal Death, Not Biological Death

Some individuals who advocate for respecting surrogate decision to continue technologic support question the biological legitimacy of the concept of BD/DNC, as the criteria used to declare BD/DNC do not require proof that all neurons in the brain have died. Patients who meet criteria for BD/DNC can have preserved neurohormonal function that allows for growth, recovery from surgery, menstruation, and even gestation of a fetus [17]. Thus, advocates of respecting surrogate decisions argue that this ambiguity legitimizes moral and religious objections given that the current legal definition of death is a social construct [12]. Though many opponents to indefinite technologic support acknowledge arguments questioning the biological validity of existing definitions and criteria of BD/DNC, they argue that from a societal perspective, a consistent definition of death is important to avoid scenarios in which patients in the same neurologic state would be considered alive in some jurisdictions and dead in others [11]. Others claim that the current standards for BD/DNC are sufficiently accepted because they indicate if not biological death, then the permanent loss of consciousness and essential personhood, which is sufficient to declare moral and legal death [18].

### Dignity and Respect

Proponents of honoring surrogate objections to discontinue technologic support argue that respect for the dignity of both the patient and family requires adhering to their wishes and beliefs about death and providing the care their surrogates state they would want [13]. Meanwhile, opponents argue that any further interventions after BD/DNC declaration constitute desecration of a deceased body’s dignity and a violation of the goals of the medical profession [14]. Staff caring for patients and families may experience moral distress if they are asked to provide care that is inconsistent with their perception of treating a patient with dignity and respect.

### Surrogate Authority

Those who favor respecting surrogate objections argue that the rights of surrogates to make decisions regarding withdrawal of technologic support after BD/DNC declaration and their beliefs about what constitutes death should not be violated [19]. Others opposing accommodations claim the rights of surrogates are not absolute, especially when their requests are contrary to the best interest of the patient, and that surrogate autonomy cannot force a medical provider to continue interventions that are inappropriate or contrary to the guiding principles of medicine [20, 21].

### Medical Mistrust

Those in favor of honoring a surrogate decision to continue technologic support claim that denying requests for ongoing technologic support increases distrust, particularly when experienced by vulnerable and historically underserved populations, as they may perceive that withdrawal of technology is being pursued to obtain organs or free up resources for other patients [22–24]. Opponents argue that allowing indefinite accommodations exacerbates inconsistency and confusion around death, paving the way for greater controversy and erosion of trust in the medical profession’s ability to accurately determine death [25].

## Discussion

**Fig. 2:**
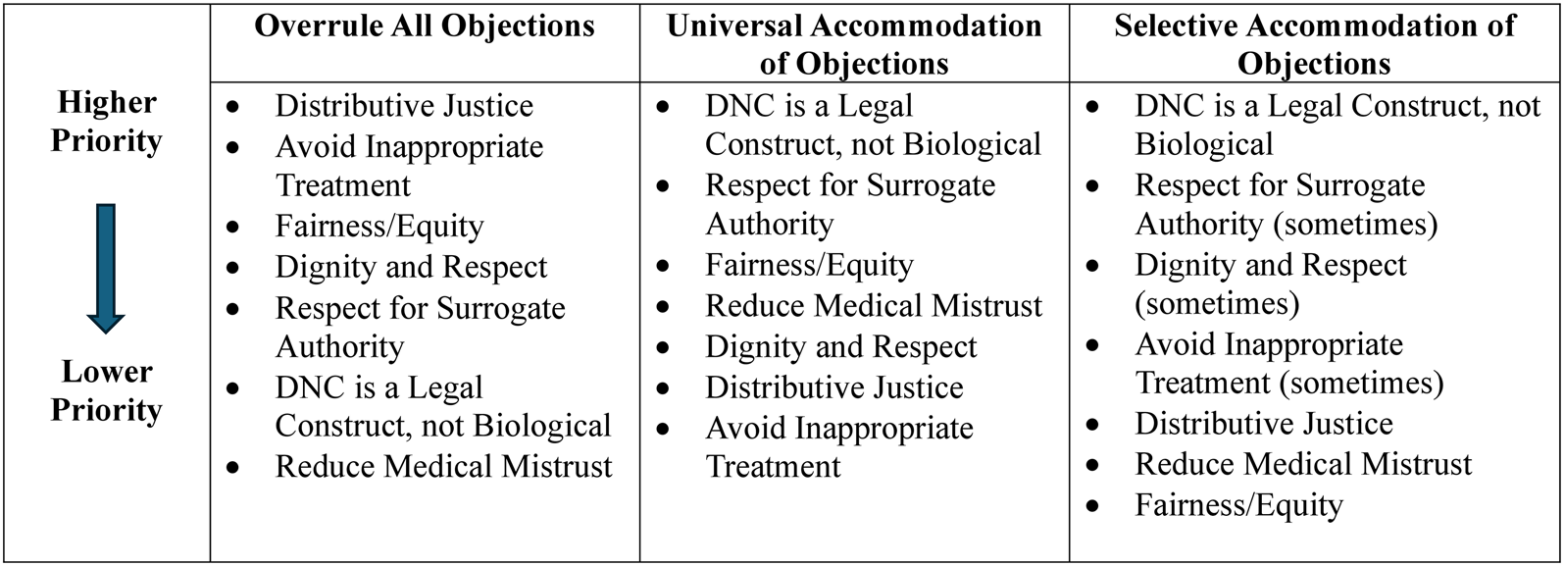
Prioritization of ethical themes in three responses to objections to removal of technologic support after brain death/death by neurologic criteria (BD/DNC)

As institutions update or construct hospital policies related to handling requests for continued technologic support after BD/DNC declaration, policy makers must recognize that some ethical principles will be prioritized over others, given they are in tension. How individual organizations choose to handle these objections may be driven by different resource constraints, varying levels of commitment to religious and philosophical differences within their community, and the degree to which they prioritize surrogate authority. We offer three general approaches for how institutions may choose to respond to requests for continued technologic support and describe the ethical principles given highest priority and those given lower priority in each scenario. When possible, we suggest ways to honor the ethical values that have lower priority in any given approach to ensure they are not entirely overlooked. Given concerns about potential unequal treatment, we encourage not only transparency of these approaches but also consistent application to avoid the appearance of, or actual, bias in treatment of patients and families. We articulate fairness/equity as a seventh relevant ethical principle (Fig. 2). Although there is also a potential impact on organ donation, which is of ethical value, there are no data to suggest that any of these approaches may have a unique impact on a surrogate’s willingness to choose organ donation at the time of death, and so we have not considered it a core value for this article’s purposes. Future studies investigating how different approaches impact organ donation are warranted. The three general approaches are as follows: (1) universal removal of technologic support irrespective of the nature of the objection, (2) universal accommodation and continued technologic support when faced with any objection, and (3) selective accommodation for some objections only.

### Universal Removal of Technologic Support Over any Surrogate Objection

We use the phrase “universal removal of technologic support” to mean overriding all objections to declaring BD/DNC and removing technologic support. Surrogates may express different reasons for their objection to removal of technologic support, including disbelief in the prognosis, a religious or philosophical objection to BD/DNC, or an emotional grief response [26]. Proponents argue that the consistency and transparency of this approach may reduce bias in policy implementation, avoiding favoritism. Universal removal of technologic support prioritizes distributive justice by freeing up resources dedicated to BD/DNC patients, allowing other patients to use those resources. Distributive justice is of variable practical importance in different hospital settings. For example, in a rural ICU with limited beds, staff, and ventilators, maintaining a BD/DNC patient on technologic support may significantly strain resources. Conversely, at a large quaternary academic medical center with a surplus of resources, care for these patients may be less burdensome, aside from ensuring adequate staffing and bed coordination. Staff morale can also be considered a scarce resource, and the impact of any approach on staff well-being and retention could be a real concern.

Universal removal of technologic support prioritizes avoiding potentially inappropriate treatments. In the United States, BD/DNC is considered legal death for patients who meet the established criteria, so providing treatment to a dead patient would be considered inappropriate. Even if transfer to a long-term care facility is a goal shared by surrogates and the ICU team, the patient’s condition may not allow it. The physiologic consequences that result from catastrophic neurologic injury in patients declared BD/DNC often lead to ongoing medical care needs that can only be provided in an ICU setting. Professional guidelines support invoking the construct of potentially inappropriate treatment in this setting and provide justification for unilateral discontinuation of technologic support [9]. The most ethically defensible approach for applying an inappropriate treatment justification would be to apply it across the entire ICU system, not just for patients who are BD/DNC. There are multiple other types of patients who may also have a very low likelihood of leaving the ICU and consuming significant resources, and this standard should apply to them as well.

The values deprioritized with universal withdrawal of technologic support are respect for surrogate authority and recognition that BD/DNC is not equivalent to biological death and that families may have differences of opinion about what constitutes death rooted in their religious, moral, or philosophical beliefs. Further, medical mistrust may be increased in this approach, as families may perceive that their values (and their loved ones) are not being respected. Institutions may also worry, given the plan to override surrogate authority, that surrogates may seek alternative avenues for their concerns to be heard and may turn to social media and recording clinical events with the intention of pressuring institutions to listen to their objections or deterring them from following through with a withdrawal.

To reduce the intensity of conflict or likelihood that the conflict will escalate to this point, opportunities to respect these lower priority ethical values should be widely instituted. These would include providing time for clinicians to understand and respond to objections from families by instituting predetermined delays in the BD/DNC evaluation process. Anecdotally, many institutions have acknowledged that they will offer some amount of time accommodating surrogate refusal of withdrawal, even if the law does not require it. If objections persist, teams could provide families options, including seeking transfer to another institution or obtaining legal counsel. Staff should be educated in the philosophical and religious differences in the understanding of death and that the legal definition of BD/DNC is considered by some to be a legal fiction or social construct rather than a biological reality [27]. Helping staff avoid judgment of surrogates who object to withdrawal of technology will also demonstrate the dignity and respect all patients and families deserve. Consideration should be given to the social and financial inequities that may make it difficult for marginalized patients to obtain transfers or legal counsel, potentially worsening mistrust. In all cases, if technologic support is universally withdrawn per hospital policy, clinicians should seek to transparently inform and partner with families as much as possible to best honor these deprioritized values.

### Universal Accommodation of Continued Technologic Support Based on Any Objection

A second potential approach is to honor all objections to BD/DNC and continue indefinite technologic support, what we call “universal accommodation.” This approach prioritizes surrogate authority and honors different beliefs about the concept of death by allowing families to unilaterally choose to continue technologic support based on their beliefs. Universal accommodation also allows for transparency and consistency in practice by treating all objections equally and may decrease medical mistrust by allowing more surrogate authority around both BD/DNC determination and organ donation. It is also possible that not consistently applying the same standard for death may lead some to question the validity of BD/DNC and may increase distrust in the concept. Providers should continue to educate families about the patient’s diagnosis and prognosis when disbelief and limitations of understanding appear to be the primary barrier to allowing removal of technology. An approach of empathy and trauma-informed care may help families who need time to adjust to the tragedy of their child’s loss and realize that they do not want to continue technologic support indefinitely. However, if objections remain intractable, patients may require additional surgeries and invasive devices, for example, tracheostomy and gastrostomy tubes, to allow eventual transfer out of the ICU, unless a long-term facility exists that will allow transfer without these. Some surgeons may object to performing procedures on patients who have met BD/DNC criteria, and therefore ensuring there are surgeons willing to perform these procedures is essential to make universal accommodation viable. In some settings, the ability to leave the ICU may be limited or nonexistent if long-term care facilities are unwilling or unable to care for patients with tenuous hemodynamics or other complex medical issues, leaving these patients in the ICU for months or years.

The values of distributive justice and inappropriate treatment are given less priority in universal accommodation, as medical resources would be used, potentially indefinitely, to support patients who might undergo further medical interventions without clear medical benefit. In facilities with limited resources, stewardship of medical supplies, beds, and staff may be strained by continuing technologic support in such cases. Insurance companies may also refuse payment for dead patients, leaving families with significant medical bills they are unable to pay. As previously described, some may argue that universal accommodation dishonors the integrity and sanctity of the deceased patient’s body by continuing potentially invasive interventions. In such cases, providers should consider resource allocation at the unit level, rather than at the individual patient level, and should consider how to best honor the dignity of the patient as technologic support continues.

### Selective Accommodation to Withdrawal of Technologic Support Based on the Type of Objection

A third approach accommodates some objections to withdrawal of technologic support after BD/DNC declaration by endorsing certain ethical concerns as more legitimate and therefore requiring respect. This approach, which we call “selective accommodation,” would involve honoring certain “principled” objections (e.g., established religious beliefs about death) and continuing technologic support indefinitely while at the same time unilaterally removing technologic support from patients whose surrogates have “informational” or “emotional” reasons for objection (e.g., disbelief in the prognosis, hope for recovery, or distrust in the medical team) [26].

Principled objections entail different beliefs about the definition of death and a surrogate’s belief that BD/DNC does not constitute true death. Because they are often rooted in a larger community with a historic tradition, principled objections may be viewed by clinicians as more legitimate than those that are informational or emotional. Historically, these objections have been given more weight by the legal system, which in a few states requires continuation of technologic support if religious or moral objections to BD/DNC exist [6]. However, in practice, surrogates may hold more than one type of objection or may initially express informational or emotional objections before progressing to principled objections if they discover (or are advised) that principled objections are more likely to be accommodated.

This final approach is ethically fraught in that it raises equity concerns and limits the transparency and consistency of policies. For example, a family may first express hope for recovery via emotional and informational objections but, when unilateral withdrawal of technology is threatened, may raise a principled objection to the legitimacy of BD/DNC. Would the hospital deprioritize the principled objection simply because it arose later? Such an approach may appear to afford less respect to surrogates with objections that are considered by the hospital to be less “legitimate.” Selective accommodation may also increase medical mistrust, especially if subjective decisions about which patients are deserving of continued organ support increase inequities for marginalized patients. Selective accommodation may also deprioritize distributive justice in permitting inequitable allocation of resources to patients with certain objections over others. This said, those opposing continued technologic support may argue that freeing up some ICU beds would place less strain on the larger medical system.

If selective accommodation is adopted, standardized adjudication of different BD/DNC objections must be implemented to best honor the deprioritized values of dignity and respect, BD/DNC as a philosophical and legal definition of death, and medical mistrust. Further, hospitals should consider who may be tasked with addressing such situations and deciding where to allocate ICU resources, such as ICU and hospital leadership, ethics consultants, and legal counsel. A standardized, transparent, process-based approach could hopefully decrease inequities. Whichever policy a hospital chooses for handling objections to BD/DNC and that they find to be most appropriate to fulfilling their mission and serving their population, following an established written hospital policy is a superior ethical solution to most physicians’ present ad hoc approach.

## Conclusions

When developing or revising hospital policies on BD/DNC, hospitals must understand the ethical values in tension with each approach. Hospitals vary in terms of the population they care for and resources at their disposal, which may impact prioritization of the ethical values at stake in these decisions. Independent of how a hospital chooses to handle requests for continued technologic support after BD/DNC declaration, some ethical values will be deprioritized. Policy makers should design systems to honor these values to the extent possible given the plurality of beliefs and experiences of the patients for whom we care.

## Supplementary Information

Below is the link to the electronic supplementary material.

Supplementary file 1 (DOCX 543 kb)
