# Supplementary material for: Prioritization of Ethical Themes When Surrogates Object to Technology Removal After Brain Death Determination
Source: Neurocrit Care. 2025 Oct 8;43(3):727–35. doi: 10.1007/s12028-025-02390-2 (PMC12647313; doi:10.1007/s12028-025-02390-2)
Supplement: Supplementary file 1 — (DOCX 543 kb) [file 12028_2025_2390_MOESM1_ESM.docx]

**Supplementary Table. Narrative review of ethics themes in objections to removal of technological support after BD/DNC declaration**

| **Ethical Value** | **Explanation** | **Articles in favor of continued technologic support in BD/DNC patients** | **Articles against continued technologic support in BD/DNC patients** | **Articles neutral to continued organ support in BD/DNC patients** |
| --- | --- | --- | --- | --- |
| **Inappropriate Treatment** | Administration of medical treatments unable to fulfill a desired physiologic effect | ^1–7^ | ^8–29^ | ^30–38^ |
| **Distributive Justice** | Equitable allocation of relatively scarce resources, including funding, medical supplies, and personnel | ^1–5,7,39,40^ | ^8,9,14,16,21,28,29,41–47^ | ^25,30,31,34,48–53^ |
| **BD/DNC as a philosophical and legal definition of death, not biological** | Neurologic death as a debated physiologic diagnosis; BD/DNC as a moral, philosophical, and legal definition of death rather than biological | ^1–3,6,7,40,54–58^ | ^10,15,21,24,28,34,46,48–51,53,59–66^ | ^19,32,33,38,67^ |
| **Dignity and Respect** | Honoring the value intrinsic to each human person; accommodating and balancing the stated perspectives of both the patient and patient’s family to best honor their needs, beliefs, and worth | ^1–5,7,39,54–56,58,68^ | ^8,9,13,16–22,27–29,42,44–46,69,70^ | ^25,37,48,50,51,61,62,71–73^ |

**Supplementary Table. Narrative review of ethics themes in objections to removal of technological support after BD/DNC declaration (continued)**

| **Ethical Value** | **Explanation** | **Articles in favor of continued technologic support in BD/DNC patients** | **Articles against continued technologic support in BD/DNC patients** | **Articles neutral to continued organ support in BD/DNC patients** |
| --- | --- | --- | --- | --- |
| **Surrogate Authority** | The right to independent decision making of surrogates to reflect a patient’s beliefs and wishes; weighed against the judgment and authority of healthcare providers in care decisions | ^1–4,7,40,54–56^ | ^8,9,11,12,14,16–19,21,23,26–28,32,33,41–47,69,70,74,75^ | ^15,25,30,34,35,37,47,49–52,59,61,71,72^ |
| **Medical Mistrust** | Lack of trust in the healthcare system and providers to care for patients’ interests; the belief that the medical system will provide differential care or act with ill-intent toward certain patients | ^5,7,55,57,68^ | ^8,9,13,14,16,18,23,28,41,43,45–47,69,70,75^ | ^34–36,51,52,59–62,71,73^ |

**References**

1. du Toit J, Miller F. The Ethics of Continued Life-Sustaining Treatment for those Diagnosed as Brain-dead. *Bioethics*. 2016;30(3):151-158. doi:10.1111/bioe.12178

2. Brown SD. Is there a place for CPR and sustained physiological support in brain-dead non-donors? *J Med Ethics*. 2017;43(10):679-683. doi:10.1136/medethics-2015-103106

3. Luce JM. Chronic disorders of consciousness following coma: Part Two: Ethical, legal, and social issues. *Chest*. 2013;144(4):1388-1393. doi:10.1378/chest.13-0428

4. Applbaum AI, Tilburt JC, Collins MT, Wendler D. A family’s request for complementary medicine after patient brain death. *JAMA*. 2008;299(18):2188-2193. doi:10.1001/jama.299.18.2188

5. Khanna R, Chaudhry MA. Discontinuation of ventilation after brain stem death: Asystole rapidly follows brain stem death. *BMJ*. 1999;319:1366-1367. doi:10.1136/bmj.319.7221.1366a

6. Miller FG. Medical futility and “brain death.” *Perspect Biol Med*. 2018;60(3):400-402. doi:10.1353/pbm.2018.0014

7. Johnson LSM. Arguments Favoring Continuation of “Organ Support” when Families Object to Declaration of Death by Neurologic Criteria. In: Lewis A, Bernat JL, eds. *Death Determination by Neurologic Criteria: Advances in Neuroethics*. Springer, Cham; 2022:467-477. doi:10.1007/978-3-031-15947-3_34

8. Barron RS. Death: Past, present, and future. *J Crit Care*. 2015;30(1):214-215. doi:10.1016/j.jcrc.2014.10.023

9. Flamm AL, Smith ML, Mayer PA. Family Members’ Requests to Extend Physiologic Support after Declaration of Brain Death: A Case Series Analysis and Proposed Guidelines for Clinical Management. *The Journal of Clinical Ethics Fall*. 2014;25(3):222-259. doi:10.1086/JCE201425307

10. De Georgia MA. History of brain death as death: 1968 to the present. *J Crit Care*. 2014;29(4):673-678. doi:10.1016/j.jcrc.2014.04.015

11. Fontugne EA. To treat or not to treat: End-of-life care, patient autonomy, and the responsible practice of medicine. *Journal of Legal Medicine*. 2014;35(4):529-538. doi:10.1080/01947648.2014.981445

12. Paola FA. The Case of Jahi McMath: Professionalism and Knowing One’s Limitations. *The Journal of Physician Assistant Education*. 2014;25(1):59-61. doi:10.1097/01367895-201425010-00011

13. Varelas PN, Abdelhak T, Hacein-Bey L. Withdrawal of life-sustaining therapies and brain death in the intensive care unit. *Semin Neurol*. 2008;28(5):726-736. doi:10.1055/s-0028-1105969

14. Cavell R. Not-for-resuscitation orders: the medical, legal and ethical rationale behind letting patients die. *J Law Med*. 2008;16(2):305-334.

15. Truog RD. End-of-life decision-making in the United States. *Eur J Anaesthesiol*. 2008;25(SUPPL. 42):43-50. doi:10.1017/S0265021507003419

16. Burck R, Anderson-Shaw L, Sheldon M, Egan EA. The Clinical Response to Brain Death: A Policy Proposal. *Ethics, and Regulation*. 2006;8(2):53-59. doi:10.1097/00128488-200604000-00008

17. Manno EM, Wijdicks EFM. The declaration of death and the withdrawal of care in the neurologic patient. *Neurol Clin*. 2006;24(1):159-169. doi:10.1016/j.ncl.2005.10.005

18. Bernat JL. Ethical Matters Medical Futility Definition, Determination, and Disputes in Critical Care. *Neurocrit Care*. 2005;2(2):198-205. doi:10.1385/Neurocrit

19. Fisher M, Raper RF. Brain stem death: managing care when accepted medical guidelines and religious beliefs are in conflict: Delay in stopping treatment can become unreasonable and unfair. *BMJ*. 2000;320:1268. doi:10.1136/bmj.320.7244.1266

20. Glannon W. Tracing the Soul: Medical Decisions at the Margins of Life. *Christ Bioeth*. 2000;6(1):49-69. doi:10.1076/1380-3603(200004)6:1;1-C;FT049

21. Fisher J. Re-examining death: against a higher brain criterlon. *Journal ofMedical Ethics*. 1999;25:473-476. doi:10.1136/jme.25.6.473

22. Michaeli D. Discontinuation of ventilation after brain stem death: Jews accept brain stem death. *BMJ*. 1999;319:1367. www.bmj.com

23. Paris JJ, Muir JC, Reardon FE. Ethical and Legal Issues in Intensive Care. *J Intensive Care Med*. 1997;12(6):298-309. doi:10.1177/088506669701200603

24. Shewmon DA. Recovery from “Brain Death”: A Neurologist’s Apologia. *Linacre Q*. 1997;64(1):30-96. doi:10.1080/20508549.1999.11878373

25. Shiner K. Medical futility: a futile concept? *Wash Lee Law Rev*. 1996;53(2):803-848. https://proxy.library.upenn.edu/login?url=https://www.proquest.com/scholarly-journals/medical-futility-futile-concept/docview/236280061/se-2?accountid=14707

26. Luce JM. Physicians do not have a responsibility to provide futile or unreasonable care if a patient or family insists. *Crit Care Med*. 1995;23(4):760-766. doi:10.1097/00003246-199504000-00027

27. Paris JJ, Bell AJ, Murphy JJ. Pediatric brain death: dead is dead. *J Perinatol*. 1995;15(1):67-70.

28. Omelianchuk A, Magnus D. Arguments Opposing Continuation of Organ Support when Families Object to Declaration of Death by Neurologic Criteria. In: Lewis A, Bernat JL, eds. *Death Determination by Neurologic Criteria: Advances in Neuroethics*. Springer, Cham; 2022:479-489. doi:10.1007/978-3-031-15947-3_35

29. Burkle CM, Sharp RR, Wijdicks EF. Why brain death is considered death and why there should be no confusion. *Neurology*. 2014;83(16):1464-1469. doi:10.1212/WNL.0000000000000883

30. Morparia K, Dickerman M, Hoehn KS. Futility: Unilateral decision making is not the default for pediatric intensivists. *Pediatric Critical Care Medicine*. 2012;13(5). doi:10.1097/PCC.0b013e31824ea12c

31. Pendleton C, Jiang B, Geocadin RG, Quinones-Hinojosa A. “Any possible restoration of function could not occur”: Harvey cushing and the early description of brain death. *World Neurosurg*. 2012;77(2):394-397. doi:10.1016/j.wneu.2011.04.016

32. Banja JD. Are Brain Dead Patients Really Dead? *J Head Trauma Rehabil*. 2009;24(2):141-144. doi:10.1097/HTR.0b013e3181a2858d

33. Miller FG, Truog RD. The incoherence of determining death by neurological criteria: A commentary on controversies in the determination of death, a white paper by the president’s council on bioethics. *Kennedy Inst Ethics J*. 2009;19(2):185-193. doi:10.1353/ken.0.0282

34. Truog R. Brain death - too flawed to endure, too ingrained to abandon. *The Journal of law, medicine & ethics*. 2007;35(2):273-281. doi:10.1111/j.1748-720X.2007.00136.x

35. DuBois JM, Anderson EE. Attitudes toward death criteria and organ donation among healthcare personnel and the general public. *Progress in transplantation*. 2006;16(1):65-73. doi:10.1177/152692480601600113

36. Youngner SJ, Arnold RM, Devita MA. When Is “Dead”? *Hastings Cent Rep*. 1999;29(6). doi:10.2307/3527866

37. Ross J, Wenger N. INSTITUTIONAL ETHICS: HOSPITAL PRACTICES AND POLICIES FOR DENYING LIFE-SUSTAINING TREATMENT. *Whittier Law Rev*. 1994;15(1):33-49. https://heinonline.org/HOL/P?h=hein.journals/whitlr15&i=43

38. Kirschen MP, Lewis A, Rubin M, Kurtz P, Greer DM. New perspectives on brain death. *J Neurol Neurosurg Psychiatry*. 2021;92(3):255-262. doi:10.1136/jnnp-2020-323952

39. Lee BM, Trowbridge A, McEvoy M, Wightman A, Kraft SA, Clark JD. Can a parent refuse the brain death examination? *Pediatrics*. 2020;145(4). doi:10.1542/peds.2019-2340

40. Muramoto O. Is informed consent required for the diagnosis of brain death regardless of consent for organ donation? *J Med Ethics*. 2021;47(12):E5. doi:10.1136/medethics-2020-106240

41. Swinburn JMA, Ali SM, Banerjee DJ, Khan ZP. Discontinuation of ventilation after brain stem death: To whom is our duty of care? *BMJ*. 1999;318:1753-1754. doi:10.1136/bmj.318.7200.1753

42. Kompanje EJO. Request for Complementary Medicine After Brain Death - Commentary. *J Am Med Assoc*. 2008;300(13):1517. doi:10.1001/jama.300.13.1517-a

43. Liao S, Ito S. Brain death: Ethical challenges to palliative care concepts of family care. *J Pain Symptom Manage*. 2010;40(2):309-313. doi:10.1016/j.jpainsymman.2010.02.016

44. Gostin LO. Legal and ethical challenges in brain death. *JAMA*. 2014;311(7):717-728. doi:10.1001/jama.2014.65

45. Lewis A, Adams N, Varelas P, Greer D, Caplan MA. Organ support after death by neurologic criteria Results of a survey of US neurologists. *Crit Care Med*. 2016;45(9):916-924. doi:10.1212/WNL.0000000000003008

46. Paris JJ, Cummings BM, Moore MP. Brain Death, Dead, and Parental Denial. *Cambridge Quarterly of Healthcare Ethics*. 2014;23(4):371-382. doi:10.1017/S0963180114000048

47. Lewis A, Adams N, Chopra A, Kirschen MP. Organ Support after Death by Neurologic Criteria in Pediatric Patients. *Crit Care Med*. 2017;45(9):e916-e924. doi:10.1097/CCM.0000000000002452

48. Singer P. PRESIDENTIAL ADDRESS: IS THE SANCTITY OF LIFE ETHIC TERMINALLY ILL? *Bioethics*. 1995;9(3):327-343. doi:10.1111/j.1467-8519.1995.tb00368.x

49. Burrows R. Removal of life support in intensive care units. *Med Law*. 1994;13(5-6):489-500. https://heinonline.org/HOL/P?h=hein.journals/mlv13&i=493

50. Truog RD. Is it time to abandon brain death? *Hastings Center Report*. 1997;27(1):29-37. doi:10.2307/3528024

51. Shah SK, Miller FG. Can we handle the truth? Legal fictions in the determination of death. *Am J Law Med*. 2010;34(4):540-585. doi:10.1177/009885881003600402

52. Belling C. The living dead. *Perspect Biol Med*. 2010;53(3):439-451. doi:10.1353/pbm.0.0168

53. Mazzola MA, Russell JA. Neurology ethics at the end of life. In: *Handbook of Clinical Neurology*. Vol 191. Elsevier B.V.; 2023:235-257. doi:10.1016/B978-0-12-824535-4.00012-4

54. Wildes KW. Death: A Persistent Controversial State. *Kennedy Inst Ethics J*. 1996;6(4):378-381. doi:10.1353/ken.1996.0046

55. Friedrich AB. More Than “Spending Time with the Body”: The Role of a Family’s Grief in Determinations of Brain Death. *J Bioeth Inq*. 2019;16(4):489-499. doi:10.1007/s11673-019-09943-z

56. Yanke G, Rady MY, Verheijde JL. Ethical and Legal Concerns With Nevada’s Brain Death Amendments. *J Bioeth Inq*. 2018;15(2):193-198. doi:10.1007/s11673-018-9852-y

57. Truog RD. Defining death: Lessons from the case of jahi McMath. *Pediatrics*. 2020;146:S75-S80. doi:10.1542/peds.2020-0818O

58. Choong KA, Rady MY. Re A (A Child) and the United Kingdom Code of Practice for the Diagnosis and Confirmation of Death: Should a Secular Construct of Death Override Religious Values in a Pluralistic Society? *HEC Forum*. 2018;30(1):71-89. doi:10.1007/s10730-016-9307-y

59. Shewmon DA. Brainstem death," “brain death” and death: a critical re- evaluation of the purported equivalence. *Issues Law Med*. 1998;14(2):125-145.

60. Whetstine LM. Biophilosophical criticisms of brain death: The need for a new paradigm. *J Crit Care*. 2014;29(5):878-880. doi:10.1016/j.jcrc.2014.06.016

61. Pope TM. Legal briefing: Brain death and total brain failure. *J Clin Ethics*. 2014;25(3):245-257. doi:10.1086/JCE201425309

62. Lewis A. End of Life THE HISTORY OF BRAIN DEATH AND HOW THE JAHI MCMATH CASE WILL IMPACT FUTURE BRAIN DEAD PATIENTS. *Medicine and Law World Association for Medical Law*. 2015;34:497-508. https://heinonline.org/HOL/P?h=hein.journals/mlv34&i=519

63. Truog RD. Defining death: making sense of the case of Jahi McMath. *JAMA - Journal of the American Medical Association*. 2018;319(18):1859-1860. doi:10.1001/jama.2018.3441

64. Rissman L, Paquette ET. Ethical and legal considerations related to disorders of consciousness. *Curr Opin Pediatr*. 2020;32(6):765-771. doi:10.1097/MOP.0000000000000961

65. Veatch RM. Would a Reasonable Person Now Accept the 1968 Harvard Brain Death Report? A Short History of Brain Death. *Hastings Center Report*. 2018;48:S6-S9. doi:10.1002/hast.943

66. Veatch RM. Killing by Organ Procurement: Brain-Based Death and Legal Fictions. *Journal of Medicine and Philosophy (United Kingdom)*. 2015;40(3):289-311. doi:10.1093/jmp/jhv007

67. Bonelli RM, Prat EH, Bonelli J. Philosophical considerations on brain death and the concept of the organism as a whole. *Psychiatr Danub*. 2009;21(1):3-8. https://www.psychiatria-danubina.com/UserDocsImages/pdf/dnb_vol21_no1/dnb_vol21_no1_3.pdf

68. Goodwin M. Revisiting Death: Implicit Bias and the Case of Jahi McMath. *Hastings Center Report*. 2018;48:S77-S80. doi:10.1177/0885066618784268

69. Kitzinger J, Kitzinger C. The “window of opportunity” for death after severe brain injury: Family experiences. *Sociol Health Illn*. 2013;35(7):1095-1112. doi:10.1111/1467-9566.12020

70. Russell JA, Epstein LG, Greer DM, Kirschen M, Rubin MA, Lewis A. Brain death, the determination of brain death, and member guidance for brain death accommodation requests. *Neurology*. 2019;92(5):228-232. doi:10.1212/WNL.0000000000006750

71. Ayeh DD, Tak HJ, Yoon JD, Curlin FA. U.S. Physicians’ Opinions about Accommodating Religiously Based Requests for Continued Life-Sustaining Treatment. *J Pain Symptom Manage*. 2016;51(6):971-978. doi:10.1016/j.jpainsymman.2015.12.337

72. Foster LW, McLellan LJ. Translating psychosocial insight into ethical discussions supportive of families in end-of-life decision-making. *Soc Work Health Care*. 2002;35(3):37-51. doi:10.1300/J010v35n03_03

73. Fainberg N, Mataya L, Kirschen M, Morrison W. Pediatric brain death certification: A narrative review. *Transl Pediatr*. 2021;10(10):2738-2748. doi:10.21037/tp-20-350

74. Gostin LO. Legal and ethical responsibilities following brain death: The McMath and Muñoz cases. *JAMA*. 2014;311(9):903-904. doi:10.1001/jama.2014.660

75. McCullough LB. Request for Complementary Medicine After Brain Death. *J Am Med Assoc*. 2008;300(13):1517. doi:10.1001/jama.300.13.1517-b
